# Longitudinal associations of housework with frailty and mortality in older adults: Singapore Longitudinal Ageing Study 2

**DOI:** 10.1186/s12877-022-03591-6

**Published:** 2022-12-13

**Authors:** Shuen Yee Lee, Ma Shwe Zin Nyunt, Qi Gao, Xinyi Gwee, Denise Qian Ling Chua, Keng Bee Yap, Shiou Liang Wee, Tze Pin Ng

**Affiliations:** 1 Health and Social Sciences Cluster, Singapore Institute of Technology, Singapore, Singapore; 2 Office of the Senior Deputy President & Provost, National University of Singapore, Singapore, Singapore; 3 National Public Health and Epidemiology Unit, National Centre for Infectious Diseases, Singapore, Singapore; 4 Department of Psychological Medicine, Yong Loo Lin School of Medicine, National University of Singapore, Level 9, NUHS Tower Block, 1E Kent Ridge Road, Singapore, 119228 Singapore; 5 Department of Geriatric Medicine, Ng Teng Fong General Hospital, Singapore, Singapore; 6 Geriatric Education and Research Institute, Singapore, Singapore

**Keywords:** Household chores, non-recreational physical activity, cancer mortality, cardiovascular mortality, all-cause mortality, multidimensional frailty, older adults, LAPAQ

## Abstract

**Background:**

Housework may provide a sustainable form of physical activity for older adults and improve health and survival outcomes. Longitudinal studies on associations between housework status over time and health outcomes are lacking. We aim to assess the longitudinal association of intensity and duration of housework with frailty and mortality outcomes.

**Methods:**

Among 3270 community-dwelling prospective cohort study participants, aged ≥55 years, data on light housework (*N*=2996) and heavy housework (*N*=3022) were available at baseline (March 6, 2009, to June 11, 2013) and follow-up at 3 to 5 years later, (January 16, 2013 to August 24, 2018). Median time spent per week on light (≥420min/week) and heavy (>0min/week) household activities at baseline and follow-up were used to categorise individuals into three groups (i) consistent low levels of housework at both baseline and follow-up, (ii) inconsistent high levels of housework at either baseline or follow-up and (iii) consistent high levels of housework at both baseline and follow-up. Baseline and follow-up frailty index >0.10, and all-cause, cancer and cardiovascular mortality from mean 9.5 years follow-up to March 31, 2021. Effect estimates were adjusted for socio-demographics, nutritional risk, lifestyle and other physical activities.

**Results:**

Overall, participants had mean [SD] age, 66.9 [7.8] years; 1916 [62.7%] were female. Participation in high levels of light and heavy housework consistently over time was associated with decreased odds of prefrailty/frailty at follow-up, [OR,0.61;95%CI,0.40–0.94] and [OR,0.56;95%CI,0.34–0.90] respectively, in the older group aged ≥65, compared to participants with consistent low levels of housework at baseline and follow-up. Sex-stratified analysis revealed an association between regular heavy housework participation and lower prevalence of prefrailty/frailty at follow-up in older men but not women [OR,0.31;95%CI,0.13–0.72]. Regular participation in high levels of light housework was associated with 41% lower risk of all-cause mortality [95%CI,0.36–0.96] in women but not in men, and 54% lower risk of cardiovascular mortality [95%CI,0.22–0.96].

**Conclusions:**

Regular participation in above average levels of light housework is associated with decreased odds of prefrailty/frailty in older adults aged ≥65 years, and all-cause mortality in older women. Heavy housework participation is associated with decreased odds of prefrailty/frailty, especially in older men aged ≥65. Housework may be a meaningful occupation for older adults and should be encouraged for health and wellbeing.

**Supplementary Information:**

The online version contains supplementary material available at 10.1186/s12877-022-03591-6.

## Introduction

Regular physical activity mitigates the risks of adverse health outcomes associated with age, including frailty, functional disabilities and mortality [1, 2]. Earlier systematic reviews show that physical activity interventions improved physical and physiological functions in older adults, such as reducing falls, improving mobility, body composition and muscle strength, and plausibly attenuate cellular ageing through increasing antioxidant capacity, improving mitochondrial density and dynamics [3, 4]. These studies suggest that physical activity is crucial in the prevention and management of ageing and its associated effects on activities of daily living and declining quality of life [5, 6]. Yet, global surveillance data across 15 years indicate that insufficient physical activity remained at a stable and high level at 27.5% [7]. Physical activity includes recreational, commuting and occupational domains. Although a large body of supportive evidence exists on the health benefits of moderate-to-vigorous physical activity, studies have largely examined the effects of leisure-time exercise, or workplace occupational activity, rather than domestic activities [8, 9].

Compared to younger adults, older adults have higher prevalence of insufficient physical activity [10, 11], and are less likely to participate in moderate-to-vigorous recreational activity, due to factors such as increased vulnerability, accessibility of facilities and safety concerns [12, 13]. Domestic activities such as housework is a form of physical activity which may provide benefits from less intensive and more sustainable regimens [14]. Promoting increased activities that fit into everyday life, including housework and home maintenance tasks, might increase physical activity adherence and improve population health, especially in older adults, as more people would be capable of meeting physical activity guidelines [15].

Housework tasks account for a significant proportion of self-reported physical activity and are a large part of everyday activities in older people, especially among women [16]. Housework is a productive activity and a component of instrumental activities of living, which are both key factors for successful ageing. Longitudinal population cohort studies have shown that housework participation at baseline is associated with decreased mortality risks in community-dwelling older adults [17–20]. While the underlying mechanisms driving the link between housework and reduced mortality is unclear, small sample cross-sectional and intervention studies suggest that housework participation is associated with improvements in physical, cognitive and executive functions [15, 21, 22] and decreased frailty [23]. Frailty, a widely recognised geriatric syndrome characterised by multidimensional function decline, is a predictor of functional disability and mortality. Such data suggests that housework, as a form of physical activity, has potential in improving health and survival outcomes [24].

However, longitudinal studies of the effects of changes in housework status over time, on frailty and mortality outcomes from large population studies are scarce. In this study, we assessed the duration and frequency of light and heavy housework performed by community-dwelling older adults in Singapore at both baseline and follow up (mean interval 4.5 years) and examined their associations with frailty, and mortality outcomes (mean 9.5 years follow-up).

## Methods

### Study design and participants

We analysed data in the Singapore Longitudinal Ageing Study (SLAS-2), an ongoing observational prospective cohort study of ageing and health transition among middle-aged and older adults in Singapore. Participants were aged ≥55 years at baseline and able to self-ambulate, excluding individuals who were unable to participate due to severe physical or mental disabilities. In the SLAS-2 cohort, 3270 residents in South-West Singapore were recruited from 6-Mar-2009 to 11-Jun-2013. Follow-up visits and assessments were conducted approximately 3–5 years later, from 16-Jan-2013 to 24-Aug-2018 for SLAS-2. Mortality from mean 9.5 years of follow-up was determined up to 31-March-2021. Details of methodology of have been described in earlier papers [25]. Ethics approval was obtained from National University of Singapore Institutional Review Board (Ref:04–140), in accordance with the relevant guidelines and regulations by the Declaration of Helsinki and the ethical principles in the Belmont Report. All participants gave written informed consent to participate in the study. Trained nurses visited participants’ homes to perform face-to-face questionnaire interviews, and clinical measurements were performed in a local study site.

### Housework and other physical activity

At baseline and follow-up, participants were asked specific questions on frequency and time spent on light and heavy household tasks, according to the Longitudinal Ageing Study Amsterdam PA questionnaire (LAPAQ) [26]. Light housework tasks included washing the dishes, dusting, making the bed, doing and hanging out the laundry, ironing, tidying up, and cooking meals. Heavy housework tasks included window cleaning, changing beddings, beating the mat, vacuuming, washing or scrubbing the floor, and chores involving sawing, carpeting, repairing or painting. The median time spent per week on household activities was used to dichotomize participants into high and low duration groups for light housework (cut-off ≥420 min/week) and heavy housework (cut-off >0 min/week) groups. The participant’s light and heavy housework status at baseline and at follow up were used to categorise individuals into three groups of (i) low levels of housework at baseline and follow-up, (ii) high levels of housework at either baseline or follow-up and (iii) high levels of housework at both baseline and follow-up. Additionally, in supplementary analyses, light housework was assigned a metabolic equivalent of task (MET) of 2.5 and heavy housework was assigned a MET of 4.0 [27]. A cut-off of ≥600MET min/week (≥150min/week of moderate-intensity PA or ≥75min/week of vigorous intensity physical activity) was used to dichotomize participants into high and low groups, according to current physical activity guidelines [28, 29]. Other non-housework physical activity domains in LAPAQ that our study participations commonly engaged in, including walking and sport, were also included in the analyses. Participants were asked questions on duration and frequency of walking outdoors and participation in sport activities (maximum of two) at baseline and follow-up, according to the LAPAQ. Changes in housework, sport and walking duration from baseline to follow-up were expressed in hours per week.

### Main outcome measures

#### Frailty

Cumulated Deficits Frailty Index at baseline and follow-up was derived from 98 possible deficits, across multiple systems and levels, including self-rated health, falls, hearing impairment, unintended weight loss, obesity, polypharmacy, activities of daily living (e.g., toilet use, feeding) instrumental activities of daily living (using telephone, travelling, shopping), metabolic diseases, history of medical conditions (e.g., eye problem, kidney failure, asthma), depression, and other mental disorders [30, 31]. For each person, a 98-dimensional vector was constructed, giving a fractional score from 0 to 1. Participants were dichotomised into robust (Frailty Index ≤0.10) and prefrail/frail groups (Frailty Index >0.10) [32].

#### Mortality

Dates of death were completely obtained from computerized national record linkage with the National Death Registry through the Singapore National Registry of Diseases Office and all-cause, cardiovascular and cancer mortality were determined.

### Covariates

Demographic data included age, sex, ethnicity and marital status; socioeconomic status was assessed by housing status (1–2-room public housing, 3-room public housing, and higher-end 4 –5-room public or private housing type) and education, categorised according to none or primary (≤6 years of schooling), secondary (7–10 years) and tertiary (≥11 years) levels. Body weight and height were measured, and body mass index (BMI) was calculated. Baseline nutritional status was assessed using the Nutrition Screening Initiative [33], and the total weighted score ranges from 0 to 21; a score of ≥6 indicates high nutritional risk, 3–5 indicates moderate nutritional risk, and 0–2 indicates low nutritional risk. At baseline, participants were asked whether they did brisk walking or other active sports (cycling, swimming, tennis, badminton, etc.) according to the following frequencies: (i) none or less than once a month, (ii) once a month to less than once a week and (iii) once a week or more. Smoking exposure was defined by self-reports of smoking history (never smoker, former smoker, and current smoker). Self-reported alcohol history (none, occasional, frequent) was also determined.

### Statistical analysis

Categorical variables are presented as numbers (percentages), and continuous variables are presented as mean (SD). Baseline differences between high and low light and heavy housework groups were examined using Mann-Whitney U test for continuous variables, and Chi-square test for categorical variables. Binomial logistic regression determined the odds ratios (ORs) and 95%CI of associations between housework groups and prefrailty/frailty at follow-up. No issues with multicollinearity were detected using tolerance and variance inflation factors. Cox regression that satisfied the proportional hazard assumption was performed to calculate hazard ratios (HRs) and 95%CI of associations between housework groups and mortality. Estimated HRs were adjusted for age, sex, ethnicity, socioeconomic status (housing type), education, brisk walking and sports activities at baseline, nutritional risk, smoking, alcohol, marital status, and ORs were adjusted additionally for baseline prefrailty/frailty status. Sub-group analyses by age (<65 years and ≥65 years) and sex were examined for prefrailty/frailty outcomes, while sex-stratified analyses were reported for mortality outcomes. Similar logistic and cox regression models were used to determine associations of changes and baseline duration (hours per week) in light and heavy housework, walking and sport activities, with prefrailty/frailty and all-cause mortality outcomes.

Data were analysed using multiple imputation given missing data due to loss to follow-up, resulting from deaths (*N*=490) and loss to contact or refusals. The latter included 1664 or 55.5% of participants for light housework status at follow-up and 1705 or 56.4% of participants for heavy housework status at follow-up. Follow-up loss of participants for frailty outcomes included 1615 or 53.9% for light housework group and 1627 or 53.8% for heavy housework group. The follow-up outcomes were complete for long-term mortality. Multiple imputation was performed using Multivariate Imputation by Chained Equations (MICE) package (version 3.12.0) [34] to create five imputed datasets for values assumed missing at random The ORs and HRs for frailty and mortality were estimated by combining the imputed datasets using Rubin’s rules [35]. All statistical analyses were performed using R version 3.6.2 (R Foundation for statistical computing, Vienna, Austria) and a value of *p*<0.05 was considered significant.

## Results

### Baseline participant characteristics

The mean (SD) age and BMI of 2996 participants with baseline data on light housework were 66.9 (7.8) and 24.1 (4.1) respectively; 1875 (62.6%) were female, 1475 (49%) lived in higher-end private housing or public housing of 4 or 5 rooms, 1872 (62.5%) had primary or lower education and 2628 (87.7%) were Chinese. The mean (SD) age and BMI of 3022 participants with baseline data on heavy housework were 66.9 (7.8) and 24.2 (4.1) respectively; 1901 (62.9%) were female, 1474 (48.8%) lived in higher-end housing, 1897 (62.8%) had primary or lower education and 2648 (87.6%) were Chinese.

Participants who engaged in high levels of light housework were more likely to be women, younger, had higher socioeconomic status, lower education, widowed, engaged in frequently in brisk walking, had lower nutritional risk, did not consume alcohol and were never smokers (Table 1). Participants who engaged in high levels of heavy housework were more likely to be women, younger, had lower BMI and education, were never smokers, married and did not engage in brisk walking (Table 1).

**Table 1:** Baseline participant demographics according to high and low light and heavy housework groups at baseline

	Light Housework			Heavy Housework		
	Low	High	*p* value	Low	High	*p* value
No. of participants	1224	1772		1659	1363	
Age, y	68 (8.4)	66.1 (7.3)	<0.001*	68.2 (8.2)	65.3 (7.1)	<0.001*
Female sex	495 (40.4)	1380 (77.9)	<0.001*	952 (57.4)	949 (69.6)	<0.001*
Body Mass Index, kg/m2	24.3 (4.1)	24 (4.1)	0.054	24.3 (4.1)	24 (4.2)	0.011*
Housing type			<0.001*			0.732
Public housing 1-2 rooms	318 (26.0)	348 (19.6)		361 (21.8)	310 (22.7)	
Public housing 3 rooms	332 (27.1)	508 (28.7)		470 (28.3)	391 (28.7)	
Other higher-end housing	567 (46.3)	908 (51.2)		819 (49.4)	655 (48.1)	
Race or ethnicity			0.673			0.132
Chinese	1070 (87.4)	1558 (87.9)		1467 (88.4)	1181 (86.6)	
Non-Chinese	153 (12.5)	211 (11.9)		189 (11.4)	181 (13.3)	
Education			<0.001*			0.024*
None or Primary	725 (59.2)	1147 (64.7)		1027 (61.9)	870 (63.8)	
Secondary	369 (30.1)	526 (29.7)		487 (29.4)	408 (29.9)	
Tertiary	123 (10.0)	95 (5.4)		139 (8.4)	79 (5.8)	
Smoking history			<0.001*			0.004*
Never	814 (66.5)	1510 (85.2)		1252 (75.5)	1093 (80.2)	
Former smoker	232 (19.0)	130 (7.3)		225 (13.6)	141 (10.3)	
Current smoker	173 (14.1)	126 (7.1)		178 (10.7)	122 (9.0)	
Alcohol			<0.001*			0.421
None	1087 (88.8)	1673 (94.4)		1523 (91.8)	1268 (93.0)	
Occasional	80 (6.5)	50 (2.8)		78 (4.7)	51 (3.7)	
Frequent	55 (4.5)	42 (2.4)		51 (3.1)	42 (3.1)	
Marital status			0.026*			0.012*
Single	122 (10.0)	143 (8.1)		136 (8.2)	129 (9.5)	
Married	799 (65.3)	1146 (64.7)		1059 (63.8)	903 (66.3)	
Divorced	77 (6.3)	92 (5.2)		88 (5.3)	85 (6.2)	
Widowed	224 (18.3)	389 (22.0)		373 (22.5)	244 (17.9)	
Sports			0.768			0.976
None	1090 (89.1)	1592 (89.8)		1487 (89.6)	1221 (89.6)	
Occasional	41 (3.3)	53 (3.0)		50 (3.0)	43 (3.2)	
Frequent	88 (7.2)	120 (6.8)		115 (6.9)	94 (6.9)	
Brisk walking			0.043*			<0.001*
None	238 (19.4)	341 (19.2)		271 (16.3)	313 (23.0)	
Occasional	150 (12.3)	167 (9.4)		219 (13.2)	107 (7.9)	
Frequent	832 (68.0)	1254 (70.8)		1161 (70.0)	937 (68.7)	
Nutritional Risk			<0.001*			0.218
Low	797 (65.1)	1309 (73.9)		1184 (71.4)	938 (68.8)	
Moderate	336 (27.5)	347 (19.6)		369 (22.2)	320 (23.5)	
High	91 (7.4)	116 (6.5)		106 (6.4)	105 (7.7)	
Light housework (min/week)	135 (106)	987 (608)	<0.001*	508 (548)	799 (683)	<0.001*
Heavy housework (min/week)	28 (95.5)	86.6 (183)	<0.001*	0 (0)	140 (207)	<0.001*

*Statistically significant at *p*<0.05

### Housework and frailty

#### Light housework

There were 746 participants who reported low level of light housework consistently at both baseline and follow-up, 1308 participants who reported high level of light housework at either baseline or follow-up, and 942 participants who reported high level of light housework at both baseline and follow-up. Respectively, the mean follow-up years for frailty was 4.4 (SD 1.9), 4.5 (SD 2.0), and 4.7 (SD 2.0). Compared with the group with consistently low levels of light housework, the group with high level of light housework at either baseline or follow-up had 26% lower odds of prefrailty/frailty at follow-up, adjusted for baseline prefrailty/frailty status, socio-demographics, lifestyle and nutritional status (Table 2). Sub-group analyses by age revealed that high levels of light housework at baseline, follow-up or both, was associated with a graded decrease in odds of prefrailty/frailty at follow-up (30–39%) in the older group aged ≥65, but not in the younger group (*P* trend=0.037) (Table 2).

**Table 2:** Associations of housework participation with prefrailty/frailty at follow up

		Total ^a^				Men ^b^				Women ^b^			
		Exposed, N	Prefrailty/ frailty, N (%)	OR (95%CI)	*p* value	Exposed, N	Prefrailty/ frailty, N (%)	OR (95%CI)	*p* value	Exposed, N	Prefrailty/ frailty N (%)	OR (95%CI)	*p* value
***Light housework (cut-off ≥420 min/week)***													
**All ages**	Low at BL & FU	746	292 (39.1)	1 (Ref)	NA	469	167 (35.6)	1 (Ref)	NA	277	125 (45.1)	1 (Ref)	NA
	High at BL or FU only	1308	450 (34.4)	0.74 (0.56-0.97)	0.031*	486	126 (25.9)	0.63 (0.38-1.04)	0.067	822	324 (39.4)	0.83 (0.52-1.33)	0.425
	High at BL & FU	942	324 (34.4)	0.66 (0.42-1.05)	0.072	166	48 (28.9)	0.67 (0.38-1.19)	0.166	776	276 (35.6)	0.70 (0.41-1.19)	0.172
	Linear trend				0.086				0.093				0.163
**<65 years** ^c^	Low at BL & FU	315	63 (20.0)	1 (Ref)	NA	192	36 (18.8)	1 (Ref)	NA	123	27 (22.0)	1 (Ref)	NA
	High at BL or FU only	602	123 (20.4)	0.74 (0.38-1.44)	0.343	192	36 (18.8)	0.72 (0.28-1.87)	0.475	410	87 (21.2)	0.81 (0.40-1.64)	0.538
	High at BL & FU	414	78 (18.8)	0.62 (0.28-1.38)	0.215	50	3 (6.0)	0.43 (0.07-2.77)	0.347	364	75 (20.6)	0.69 (0.31-1.56)	0.355
	Linear trend				0.226				0.303				0.357
**≥65 years** ^c^	Low at BL & FU	431	229 (53.1)	1 (Ref)	NA	277	131 (47.3)	1 (Ref)	NA	154	98 (63.6)	1 (Ref)	NA
	High at BL or FU only	706	327 (46.3)	0.70 (0.52-0.94)	0.017*	294	90 (30.6)	0.60 (0.32-1.10)	0.091	412	237 (57.5)	0.79 (0.47-1.30)	0.345
	High at BL & FU	528	246 (46.6)	0.61 (0.40-0.94)	0.028*	116	45 (38.8)	0.67 (0.35-1.30)	0.226	412	201 (48.8)	0.63 (0.39-1.03)	0.064
	Linear trend				0.037*				0.086				0.072
***Heavy housework (cut-off >0 min/week)***													
**All ages**	Low at BL & FU	1144	481 (42.0)	1 (Ref)	NA	469	171 (36.5)	1 (Ref)	NA	675	310 (45.9)	1 (Ref)	NA
	High at BL or FU only	1300	463 (35.6)	0.75 (0.55-1.00)	0.053	470	132 (28.1)	0.67 (0.46-0.98)	0.041*	830	331 (39.9)	0.80 (0.52-1.21)	0.264
	High at BL & FU	578	176 (30.4)	0.60 (0.41-0.89)	0.013*	182	42 (23.1)	0.40 (0.20-0.81)	0.014*	396	134 (33.8)	0.72 (0.43-1.20)	0.192
	Linear trend				0.009*				0.002*				0.164
**<65 years** ^c^	Low at BL & FU	465	111 (23.9)	1 (Ref)	NA	186	41 (22.0)	1 (Ref)	NA	279	70 (25.1)	1 (Ref)	NA
	High at BL or FU only	594	132 (22.2)	0.65 (0.40-1.08)	0.093	174	31 (17.8)	0.64 (0.28-1.46)	0.281	420	101 (24.0)	0.65 (0.36-1.17)	0.143
	High at BL & FU	280	47 (16.8)	0.56 (0.34-0.93)	0.026*	73	10 (13.7)	0.50 (0.12-2.02)	0.309	207	37 (17.9)	0.57 (0.28-1.16)	0.114
	Linear trend				0.011*				0.187				0.092
**≥65 years** ^c^	Low at BL & FU	679	370 (54.5)	1 (Ref)	NA	283	130 (45.9)	1 (Ref)	NA	396	240 (60.6)	1 (Ref)	NA
	High at BL or FU only	706	331 (46.9)	0.73 (0.51-1.05)	0.085	296	101 (34.1)	0.61 (0.35-1.06)	0.076	410	230 (56.1)	0.81 (0.46-1.40)	0.41
	High at BL & FU	298	129 (43.3)	0.56 (0.34-0.90)	0.020*	109	32 (29.4)	0.31 (0.13-0.72)	0.009*	189	97 (51.3)	0.74 (0.37-1.50)	0.376
	Linear trend				0.013*				0.001*				0.328

^a^ Adjusted for baseline prefrailty/frailty status, age, sex, ethnicity, socioeconomic status (housing type), education, brisk walking and sports activities at baseline, nutritional risk, smoking, alcohol, marital status

^b^ Adjusted for baseline prefrailty/frailty status, age, ethnicity, socioeconomic status (housing type), education, brisk walking and sports activities at baseline, nutritional risk, smoking, alcohol, marital status

^c^ Age was excluded from model adjustments

*BL* Baseline, *FU* Follow-up, *Ref* Reference odds ratio of 1.00, *OR* Odds Ratio, *CI* Confidence Interval

*Statistically significant at *p*<0.05

#### Heavy housework

There were 1144 participants who reported low level of heavy housework at baseline and follow-up, 1300 participants who reported high level of light housework at either baseline or follow-up, and 578 participants who reported high level of light housework at both baseline and follow-up. Compared to participants who consistently did low levels of heavy housework, participation in high levels of heavy housework consistently at both baseline and follow-up showed decreased odds of prefrailty/frailty at follow-up in older adults [adjusted OR, 0.60; 95%CI, 0.41–0.89; *P* trend=0.009], consistently in both younger and older groups (Table 2). In sex-stratified analyses, the results robustly show that men in the older group who consistently engaged in high levels of heavy housework at baseline and follow-up had 69% decreased prevalence of prefrailty/frailty at follow-up [95%CI, 0.13–0.72; *P* trend=0.001] (Table 2).

Similar trends were observed when stratified according to physical activity guidelines of ≥600 MET min/week attained solely through housework. Consistent engagement in high levels of housework at baseline and follow-up was generally associated with lower odds of prefrailty/frailty [adjusted OR, 0.48; 95%CI, 0.30–0.77], especially among men in the older group [adjusted OR, 0.46; 95%CI, 0.25–0.87] (eTable 1).

### Housework and all-cause mortality

Participants with high levels of housework (≥600 MET min/week), at both baseline and follow-up, showed a 39% lower mortality risk in the fully adjusted model [95%CI, 0.47–0.79], regardless of sex (eTable 2).

#### Light housework

The mean follow-up years for mortality among participants with a low level of light housework at baseline and follow-up, a high level of light housework at either baseline or follow-up, and a high level of light housework at both baseline and follow-up were 8.9 (SD 2.7), 9.6 (SD 2.3) and 9.9 (SD 2.0) respectively. Compared with a consistent low level of light housework at both baseline and follow-up, participation in high levels of light housework showed a 43–64% lower mortality risk in crude analysis. Participation in a high level of light housework at both baseline and follow up showed a 43% lower mortality risk after adjusting for age, sex and ethnicity [HR, 0.57; 95%CI ,0.36–0.91] and 42% lower mortality risk in the fully adjusted model [HR, 0.58; 95%CI, 0.36–0.94] (Table 3). Stratified analyses by sex revealed that these associations were statistically significant in women [adjusted HR, 0.59; 95%CI, 0.36–0.96; *P* trend=0.027] but not in men [adjusted HR, 0.62; 95%CI, 0.34–1.14; *P* trend=0.082] (Table 3).

**Table 3:** Associations of longitudinal changes in housework participation with all-cause mortality

		Exposed	Deaths		Model 1		Model 2		Model 3	
		*person-years*	*N*	*Per 100 person-years*	HR (95%CI)	*p* value	HR (95%CI)	*p* value	HR (95%CI)	*p* value
***Light housework (cut-off ≥420 min/week)***										
**Total**	Low at BL & FU	7050	208	2.95	1 (Ref)	NA	1 (Ref)	NA	1 (Ref)	NA
	High at BL or FU only	11904	189	1.59	0.57 (0.39-0.82)	0.007*	0.76 (0.53-1.09)	0.123	0.77 (0.55-1.08)	0.121
	High at BL & FU	9386	93	0.99	0.36 (0.24-0.54)	<0.001*	0.57 (0.36-0.91)	0.023*	0.58 (0.36-0.94)	0.032*
	Linear trend, p value					<0.001*		0.025*		0.030*
**Men** ^a^	Low at BL & FU	4276	154	3.6	1 (Ref)	NA	1 (Ref)	NA	1 (Ref)	NA
	High at BL or FU only	4187	96	2.29	0.71 (0.43-1.18)	0.159	0.73 (0.48-1.11)	0.124	0.72 (0.49-1.07)	0.099
	High at BL & FU	1646	34	2.07	0.63 (0.36-1.11)	0.104	0.65 (0.36-1.18)	0.141	0.62 (0.34-1.14)	0.117
	Linear trend, p value					0.088		0.108		0.082
**Women** ^a^	Low at BL & FU	2774	54	1.95	1 (Ref)	NA	1 (Ref)	NA	1 (Ref)	NA
	High at BL or FU only	7717	93	1.21	0.60 (0.42-0.87)	0.008*	0.82 (0.49-1.36)	0.419	0.86 (0.51-1.46)	0.560
	High at BL & FU	7740	59	0.76	0.41 (0.27-0.63)	<0.001*	0.55 (0.35-0.87)	0.013*	0.59 (0.36-0.96)	0.033*
	Linear trend, p value					<0.001*		0.010*		0.027*
***Heavy housework (cut-off >0 min/week)***										
**Total**	Low at BL & FU	10651	260	2.44	1 (Ref)	NA	1 (Ref)	NA	1 (Ref)	NA
	High at BL or FU only	12728	185	1.45	0.64 (0.40-1.01)	0.055	0.81 (0.50-1.32)	0.336	0.78 (0.48-1.27)	0.259
	High at BL & FU	5202	52	1.00	0.44 (0.17-1.12)	0.073	0.67 (0.25-1.83)	0.357	0.64 (0.22-1.81)	0.317
	Linear trend, p value					0.057		0.327		0.282
**Men** ^a^	Low at BL & FU	4345	160	3.68	1 (Ref)	NA	1 (Ref)	NA	1 (Ref)	NA
	High at BL or FU only	4171	94	2.25	0.71 (0.36-1.40)	0.261	0.79 (0.39-1.61)	0.45	0.75 (0.38-1.48)	0.339
	High at BL & FU	1588	29	1.83	0.50 (0.19-1.31)	0.133	0.64 (0.23-1.74)	0.32	0.60 (0.21-1.68)	0.273
	Linear trend, p value					0.155		0.334		0.277
**Women** ^a^	Low at BL & FU	6306	100	1.59	1 (Ref)	NA	1 (Ref)	NA	1 (Ref)	NA
	High at BL or FU only	8557	91	1.06	0.64 (0.38-1.07)	0.084	0.84 (0.51-1.37)	0.443	0.79 (0.46-1.33)	0.334
	High at BL & FU	3615	23	0.64	0.47 (0.20-1.15)	0.087	0.72 (0.26-1.97)	0.465	0.64 (0.22-1.92)	0.363
	Linear trend, p value					0.029*		0.392		0.295

Model 1: Unadjusted; Model 2: Adjusted for age, sex, ethnicity; Model 3: Adjusted for age, sex, ethnicity, socioeconomic status (housing type), education, brisk walking and sports activities at baseline, nutritional risk, smoking, alcohol, marital status.

^a^ Sex was excluded from model adjustments.

*BL* Baseline, *FU* Follow-up, *Ref* Reference hazard ratio of 1.00, *HR* Hazard Ratio, *CI* Confidence Interval

*Statistically significant at *p*<0.05

#### Heavy housework

Similar trends of association were observed with heavy housework, although they were not statistically significantly in the fully adjusted model. However, in sensitivity analyses in which we dichotomised the level of heavy housework by the highest quartile (≥60 min/week) of heavy housework, we observed that regardless of sex, heavy housework at either baseline or follow-up was associated with 29% lower mortality risk in the fully adjusted model [95%CI, 0.56–0.91], compared to participants who did <60 min/week of heavy housework at both baseline and follow-up (eTable 3).

### Housework, cancer mortality and cardiovascular mortality

#### Light housework

In fully adjusted models, regardless of sex, similar trends of association were observed for both cancer and cardiovascular mortality, although the risk estimates were statistically significant only for high levels of light housework at both baseline and follow-up associated with 54% lower risk of cardiovascular mortality [95%CI, 0.22–0.96] (Table 4).

**Table 4:** Associations of housework participation with cancer and cardiovascular mortality

	Total ^a^					Men ^b^					Women ^b^				
	Exposed	Deaths				Exposed	Deaths				Exposed	Deaths			
	*Person-years*	*N*	*Per 100 person-years*	HR (95%CI)	*P* value	*Person-years*	*N*	*Per 100 person-years*	HR (95%CI)	*P* value	*Person-years*	*N*	*Per 100 person-years*	HR (95%CI)	*P* value
**Cancer mortality**															
***Light housework (cut-off ≥420 min/week)***															
Low at BL & FU	6981	63	0.9	1 (Ref)	NA	4237	47	1.11	1 (Ref)	NA	2744	16	0.58	1 (Ref)	NA
High at BL/FU only	11926	57	0.48	0.73 (0.48-1.13)	0.156	4219	28	0.66	0.69 (0.39-1.21)	0.184	7707	29	0.38	0.79 (0.39-1.58)	0.488
High at BL & FU	9434	26	0.28	0.42 (0.16-1.12)	0.077	1654	9	0.54	0.42 (0.14-1.22)	0.103	7780	17	0.22	0.44 (0.14-1.37)	0.138
Linear trend, p value					0.051					0.071					0.127
***Heavy housework (cut-off >0 min/week)***															
Low at BL & FU	10575	75	0.71	1 (Ref)	NA	4295	47	1.09	1 (Ref)	NA	6280	28	0.45	1 (Ref)	NA
High at BL/FU only	12761	58	0.45	0.79 (0.30-2.06)	0.563	4183	28	0.67	0.71 (0.26-1.92)	0.435	8578	30	0.35	0.88 (0.27-2.93)	0.803
High at BL & FU	5246	12	0.23	0.65 (0.11-4.02)	0.563	1625	7	0.43	0.64 (0.10-3.99)	0.564	3620	5	0.14	0.66 (0.06-6.83)	0.648
Linear trend, p value					0.532					0.447					0.683
**Cardiovascular mortality**															
***Light housework (cut-off ≥420 min/week)***															
Low at BL & FU	6992	73	1.04	1 (Ref)	NA	4263	54	1.27	1 (Ref)	NA	2729	19	0.7	1 (Ref)	NA
High at BL/FU only	11898	55	0.46	0.68 (0.37-1.25)	0.191	4185	30	0.72	0.62 (0.33-1.18)	0.134	7714	25	0.32	0.84 (0.26-2.64)	0.725
High at BL & FU	9450	27	0.29	0.46 (0.22-0.96)	0.041*	1662	8	0.48	0.55 (0.19-1.60)	0.244	7788	19	0.24	0.51 (0.17-1.55)	0.208
Linear trend, p value					0.051					0.118					0.190
***Heavy housework (cut-off >0 min/week****)*															
Low at BL & FU	10622	81	0.76	1 (Ref)	NA	4334	51	1.18	1 (Ref)	NA	6288	30	0.48	1 (Ref)	NA
High at BL/FU only	12680	59	0.47	0.82 (0.24-2.88)	0.694	4149	31	0.75	0.79 (0.14-4.45)	0.717	8531	28	0.33	0.91 (0.31-2.72)	0.844
High at BL & FU	5280	17	0.32	0.50 (0.03-8.27)	0.517	1621	10	0.62	0.37 (0.01-13.70)	0.479	3659	7	0.19	0.66 (0.05-8.70)	0.676
Linear trend, p value					0.535					0.510					0.711

^a^ Adjusted for age, sex, ethnicity, socioeconomic status (housing type), education, brisk walking and sports activities at baseline, nutritional risk, smoking, alcohol, marital status

^b^ Adjusted for age, ethnicity, socioeconomic status (housing type), education, brisk walking and sports activities at baseline, nutritional risk, smoking, alcohol, marital status

*BL* Baseline, *FU* Follow-up, *Ref* Reference hazard ratio of 1.00, *HR* Hazard Ratio, *CI* Confidence Interval

*Statistically significant at *p*<0.05

#### Heavy housework

Likewise, similarly suggestive trends of association were observed for both cancer and cardiovascular mortality, although the risk estimates were statistically non-significant (Table 4).

### Baseline level and changes in housework and other physical activity

Both baseline level and changes in light housework hours from baseline to follow-up were associated with lower odds of all-cause mortality (Table 5). An increase in sport participation from baseline to follow-up (hours per week) was associated with 6–7% lower risks of prefrailty/frailty at follow-up (Table 5). Baseline sport participation in hours per week was additionally associated with lower odds of all-cause mortality [adjusted HR, 0.90; 95%CI, 0.85–0.97] (Table 5). Baseline and changes in walking hours were not associated with prefrailty/frailty or mortality (Table 5).

**Table 5:** Associations of changes in other physical activity with prefrailty/frailty and all-cause mortality

	Prefrailty/Frailty ^a^		All-cause mortality ^b^	
	OR (95%CI)	*P* value	HR (95%CI)	*P* value
*Light housework and other physical activity from LAPAQ*				
Changes in light housework (hours/week)	0.99 (0.97-1.02)	0.482	0.98 (0.96-0.99)	0.003*
Baseline light housework (hours/week)	0.98 (0.96-1.01)	0.261	0.97 (0.95-0.99)	<0.001*
Changes in walking (hours/week)	0.98 (0.95-1.01)	0.149	0.99 (0.93-1.05)	0.584
Baseline walking (hours/week)	0.98 (0.94-1.03)	0.330	0.99 (0.94-1.05)	0.667
Changes in sport (hours/week)	0.94 (0.90-0.98)	0.006*	0.94 (0.79-1.13)	0.433
Baseline sport (hours/week)	0.89 (0.84-0.95)	0.001*	0.88 (0.74-1.05)	0.124
*Heavy housework and other physical activity from LAPAQ*				
Changes in heavy housework (hours/week)	1.02 (0.97-1.07)	0.522	1.03 (0.97-1.10)	0.261
Baseline heavy housework (hours/week)	0.94 (0.85-1.04)	0.220	1.02 (0.95-1.09)	0.605
Changes in walking (hours/week)	0.99 (0.96-1.01)	0.229	0.98 (0.93-1.03)	0.387
Baseline walking (hours/week)	0.99 (0.96-1.02)	0.472	0.98 (0.94-1.02)	0.300
Changes in sport (hours/week)	0.93 (0.88-0.98)	0.011*	0.97 (0.92-1.01)	0.167
Baseline sport (hours/week)	0.88 (0.82-0.94)	<0.001*	0.90 (0.85-0.97)	0.004*

^a^ Adjusted for age, sex, ethnicity, socioeconomic status (housing type), education, nutritional risk, smoking, alcohol, marital status and baseline prefrailty/frailty status

^b^ Adjusted for age, sex, ethnicity, socioeconomic status (housing type), education, nutritional risk, smoking, alcohol, marital status

*OR* Odds Ratio, *HR* Hazard Ratio, *CI* Confidence Interval, *LAPAQ* Longitudinal Ageing Study Amsterdam Physical Activity Questionnaire

*Statistically significant at p<0.05

## Discussion

We show that in older adults aged ≥65 years, participants who consistently engaged in high levels of light or heavy housework over time were significantly less likely (~40%) to become prefrail/frail at follow-up. This is in agreement with a previous longitudinal study in older Chinese adults which showed that housework participation at baseline was independently associated with lower risk of incident physical phenotype of frailty at follow-up [36]. We used a different measure of frailty (Frailty Index) which represents a broader construct of frailty that reflects multisystem functional and physiological decline, and which includes psychological, biological and social domains. The Frailty Index has been consistently shown to predict adverse health outcomes, including falls, cognitive impairment, multimorbidity, disability, impaired quality of life, and mortality [37].

Notably, the associations between high levels of heavy housework and decreased frailty were most strongly evident in older men but not in women. A previous cross-sectional accelerometer study also showed that moderate-to-vigorous physical activity was more consistently associated with frailty in older men than women [38]. While the underlying mechanisms driving the sex-specific differences are not known, it is plausible that men have longer sedentary time than women, suggesting that housework of higher intensities might contribute to meeting physical activity guidelines and counteracting the adverse effects of sedentary behaviours for beneficial health outcomes in men [39, 40]. Given that housework is a form of physical activity, these results collectively suggest that higher intensities of housework might be protective of frailty in men, suggesting that sex-specific physical activity interventions and guidelines should be considered.

Among the younger group of older adults aged 55–64 years, regular participation in heavy but not light housework was associated with lower odds of prefrailty/frailty. Systematic reviews and randomized controlled trials have also shown that compared to low-intensity exercises, exercise interventions of moderate-to-high intensity were more effective in improving physical function and disability among frail older adults [41, 42]. Our results suggest that similar to exercise, a dose-response effect likely exists for housework and associated reduction in frailty risk, especially in younger group of community-dwelling older adults.

In this study, consistently engaging in high levels of light housework over time was associated with lower risk of all-cause and cardiovascular mortality in older adults, especially in women. In contrast, an earlier study showed that compared to those who did not engage in any housework, participation in heavy but not light housework at baseline was associated with reduced all-cause mortality and cancer deaths in older Chinese men but not women [19]. Yet, in agreement with our study, other studies also reported that housework was associated with decreased risk of all-cause mortality in Spanish, Chinese and Mexican American older adults aged 60–75 and older [17, 18, 20]. The disparity in findings could be due to differences in study design. Heterogeneity in the assessment, type, intensity and duration of housework classification exists across studies, which might also explain the differences in findings. Housework is a productive physical activity that may enhance sense of meaning and purpose in life, plausibly conferring survival benefits through psychosocial and biological pathways, such as anti-inflammatory mechanisms [43, 44]. Future studies should better elucidate the underlying mechanisms of housework with mortality.

This study extends upon earlier findings by showing that light housework consistently performed over time is associated with favourable survival outcomes in older persons. These findings have important implications for policy and practice, as housework accounted for a significant proportion of self-reported moderate-to-vigorous-intensity physical activity among older adults, resulting in more older adults meeting physical activity guidelines through housework than recreational activities [15, 16]. It is plausible that as older adults transition into different levels of care needs, they may lose opportunities for regular leisure time physical activity [13]. Interestingly, household but not recreational physical activity has been reported to be associated positively with brain volume, specifically grey matter volume, in cognitively unimpaired older adults [22]. Time spent in light physical activity and daily step count were also associated positively with cognitive performance and quality of life and negatively with the risk of depression among nursing home residents [45]. These results collectively suggest that housework and other light physical activities could be beneficial for older adults with varying health status, for rehabilitation and maintaining function [46]. Housework can be executed regularly even at light intensities and incorporated into daily lifestyle through domestic duties for health benefits, especially in older adults.

This population-based cohort study recruited a large sample of community-dwelling older adults and demonstrated, for the first time, the impact of housework participation over time on subsequent frailty and mortality. As it is possible that housework activities could change over time, potentially influencing the effect estimates, we measured housework activities at both baseline and follow-up. This is a strength in this study. Inaccurate reporting of housework exposure due to recall bias associated with self-reported information was ameliorated by grouping participants with consistently below average (reference group) and consistently above average housework exposure (third group) at both baseline and follow up. On the other hand, the middle group of housework exposure represents a heterogeneous group of individuals, which included those with inconsistent recall of housework participation. In addition, those who reported above average (high) housework at baseline and below average (low) housework at follow-up may represent those with declining health, while those who reported housework exposure vice versa, may represent those who became more active. We considered sub-grouping this middle category of those with improving or reducing housework exposure, as such analysis may provide more granular information on their associations with subsequent adverse health outcomes. However, such multiple sub-grouping of housework exposure over time is also problematic. Granular information on the proximity between baseline and follow up in transition of housework exposure was limited by the 3 to 5 years follow up interval. For example, a participant who reported above average (high) housework at follow-up may have done so for only a short while prior to follow-up interview, and this might not provide meaningful observations about its association with subsequent health outcomes. We additionally used changes in housework duration from baseline to follow-up, in hours per week as a continuous variable, and such sensitivity analyses support a robust association of housework with favourable health outcomes.

Although the LAPAQ used in this study is valid and reliable, we are unaware of earlier studies that have validated the housework subscale [26]. The median time spent per week on household activities was used to dichotomise participants into high and low groups in this study, which is an arbitrary cut-off. More research is required to provide recommendations and guidelines for housework participation and to determine its clinical relevance. The number of cancer and cardiovascular deaths were small, especially in women, limiting the power of the study to demonstrate associations between housework and cause-specific mortality. Although we controlled for confounding by known risk factors including other physical activities such as sports and brisk walking at baseline, residual confounding by unidentified factors remains possible. The findings in this Asian community-dwelling population may not be generalizable to other populations or individuals, such as institutionalised older adults in nursing homes. There was non-trivial loss of participants at follow-up, and participants who were not reassessed at follow-up were observed to be more frail and had poorer health outcomes at baseline interview. As complete case analysis could plausibly underestimate the actual associations of housework with frailty and mortality, we used multiple imputation techniques to generate more unbiased estimates. Future intervention studies should investigate the long-term effects of housework on health outcomes to establish causality.

Participants who consistently engaged in high levels of housework are likely to be a self-selected group with higher functional ability, and conversely, their peers with declining function tend to not engage in housework activities. In this study, we found that regardless of the level of housework participation, ~70% of older adults engaged frequently in brisk walking and ~7% in sports, suggesting similar functional ability between high and low housework groups. Our data show that indeed other forms of physical activity including sport participation might be beneficial in reducing risk of frailty and mortality in older adults. While it is crucial that older adults engage in sustainable and meaningful physical activities, such as sports, depending on preferences, abilities and needs, housework may be an important form of physical activity that should not be ignored.

## Conclusions

In this cohort study of community-dwelling older adults in Singapore, regular participation in above average levels of light housework over time was associated with 39% decreased odds of prefrailty/frailty, 42% lower risk of all-cause mortality, especially in women, and 54% lower risk of cardiovascular mortality. Consistent heavy housework participation over time was associated with 44% decreased odds of prefrailty/frailty, especially in men. Older adults should be encouraged to participate in housework as a meaningful occupation to continue, even with advancing age, to sustain health and wellbeing.

## Supplementary Information

**Additional file 1: eTable 1.** Associations of participation in housework dichotomised by MET min/week with prefrailty/frailty at follow up. **eTable 2.** Associations of participation in housework dichotomised by MET min/week with all-cause mortality. **eTable 3.** Associations of participation in housework dichotomised by top quartile duration (min/week) with all-cause mortality.

## Data Availability

The datasets used and/or analysed during the current study are available from the corresponding author on reasonable request.

## Abbreviations

HR: Hazard Ratio
LAPAQ: Longitudinal Ageing Study Amsterdam Physical Activity Questionnaire
MICE: Multivariate Imputation by Chained Equations
OR: Odds Ratio
SLAS: Singapore Longitudinal Ageing Study
