# Supplementary material for: Longitudinal associations of housework with frailty and mortality in older adults: Singapore Longitudinal Ageing Study 2
Source: BMC Geriatr. 2022 Dec 13;22:962. doi: 10.1186/s12877-022-03591-6 (PMC9749321; doi:10.1186/s12877-022-03591-6)
Supplement: Supplementary file 1 — Additional file 1: eTable 1. Associations of participation in housework dichotomised by MET min/week with prefrailty/frailty at follow up. eTable 2. Associations of participation in housework dichotomised by MET min/week with all-cause mortality. eTable 3. Associations of participation in housework dichotomised by top quartile duration (min/week) with all-cause mortality. [file 12877_2022_3591_MOESM1_ESM.docx]

| **eTable 1.** Associations of housework participation dichotomised by MET min/week with prefrailty/frailty at follow up | | | | | | | | | | | | | | | | |
| --- | --- | --- | --- | --- | --- | --- | --- | --- | --- | --- | --- | --- | --- | --- | --- | --- |
|  |  | Total ^a^ | | | |  | Men ^b^ | | | |  | Women ^b^ | | | | |
|  |  | Exposed,  N | Prefrailty/ frailty, N (%) | OR (95%CI) | p value |  | Exposed,  N | Prefrailty/ frailty, N (%) | OR (95%CI) | p value |  | Exposed, N | | Prefrailty/ frailty N (%) | OR (95%CI) | p value |
| ***Housework cut-off ≥600 MET min/week*** | | | |  |  |  |  |  |  |  |  |  | |  |  |  |
| **All ages** | Low at BL & FU | 529 | 214 (40.5) | 1 (Ref) | NA |  | 343 | 117 (34.1) | 1 (Ref) | NA |  | 186 | 97 (52.2) | | 1 (Ref) | NA |
|  | High at BL or FU only | 1158 | 410 (35.4) | 0.69 (0.50-0.97) | 0.034* |  | 451 | 126 (27.9) | 0.60 (0.34-1.06) | 0.076 |  | 707 | 284 (40.2) | | 0.80 (0.50-1.27) | 0.335 |
|  | High at BL & FU | 1277 | 430 (33.7) | 0.48 (0.30-0.77) | 0.006* |  | 310 | 73 (23.5) | 0.47 (0.26-0.83) | 0.011* |  | 967 | 357 (36.9) | | 0.53 (0.30-0.93) | 0.028* |
|  | Linear trend |  |  |  | 0.005* |  |  |  |  | 0.013* |  |  |  | |  | 0.014* |
| **<65 years** ^c^ | Low at BL & FU | 204 | 42 (20.6) | 1 (Ref) | NA |  | 133 | 25 (18.8) | 1 (Ref) | NA |  | 71 | 17 (23.9) | | 1 (Ref) | NA |
|  | High at BL or FU only | 565 | 121 (21.4) | 0.68 (0.38-1.23) | 0.194 |  | 192 | 30 (15.6) | 0.65 (0.30-1.43) | 0.284 |  | 373 | 91 (24.4) | | 0.72 (0.29-1.78) | 0.465 |
|  | High at BL & FU | 548 | 115 (21.0) | 0.39 (0.17-0.95) | 0.039* |  | 102 | 18 (17.6) | 0.35 (0.07-1.74) | 0.176 |  | 446 | 97 (21.7) | | 0.42 (0.15-1.20) | 0.099 |
|  | Linear trend |  |  |  | 0.034* |  |  |  |  | 0.152 |  |  |  | |  | 0.034* |
| **≥65 years** ^c^ | Low at BL & FU | 325 | 172 (52.9) | 1 (Ref) | NA |  | 210 | 92 (43.8) | 1 (Ref) | NA |  | 115 | 80 (69.6) | | 1 (Ref) | NA |
|  | High at BL or FU only | 593 | 289 (48.7) | 0.68 (0.43-1.07) | 0.090 |  | 259 | 96 (37.1) | 0.60 (0.29-1.23) | 0.146 |  | 334 | 193 (57.8) | | 0.80 (0.46-1.38) | 0.414 |
|  | High at BL & FU | 729 | 315 (43.2) | 0.49 (0.29-0.83) | 0.012* |  | 208 | 55 (26.4) | 0.46 (0.25-0.87) | 0.019* |  | 521 | 260 (49.9) | | 0.55 (0.28-1.06) | 0.072 |
|  | Linear trend |  |  |  | 0.009* |  |  |  |  | 0.019* |  |  | |  |  | 0.049* |
| ^a^ Adjusted for baseline prefrailty/frailty status, age, sex, ethnicity, socioeconomic status (housing type), education, brisk walking and sports activities at baseline, nutritional risk, smoking, alcohol, marital status  ^b^ Adjusted for baseline prefrailty/frailty status, age, ethnicity, socioeconomic status (housing type), education, brisk walking and sports activities at baseline, nutritional risk, smoking, alcohol, marital status  ^c^ Age was excluded from model adjustments  BL=Baseline, FU=Follow-up, MET=Metabolic equivalent of task, Ref=Reference odds ratio of 1.00, OR=Odds Ratio, CI=Confidence Interval; *Statistically significant at p<0.05 | | | | | | | | | | | | | | | | |

| **eTable 2.** Associations of participation in housework dichotomised by MET min/week with all-cause mortality | | | | | | | | | | | | | |
| --- | --- | --- | --- | --- | --- | --- | --- | --- | --- | --- | --- | --- | --- |
|  |  | Exposed | Deaths | *Per 100 person-years* |  | Model 1 |  |  | Model 2 |  |  | Model 3 |  |
|  |  | *person-years* | *N* |  |  | HR (95%CI) | p value |  | HR (95%CI) | p value |  | HR (95%CI) | p value |
| ***Housework cut-off ≥600 MET min/week*** | | | |  |  |  |  |  |  |  |  |  |  |
| **Total** | Low at BL & FU | 4486 | 135 | 3.01 |  | 1 (Ref) | NA |  | 1 (Ref) | NA |  | 1 (Ref) | NA |
|  | High at BL/FU only | 11436 | 200 | 1.75 |  | 0.53 (0.40-0.70) | <0.001* |  | 0.77 (0.59-1.00) | 0.051 |  | 0.75 (0.54-1.02) | 0.065 |
|  | High at BL & FU | 12135 | 147 | 1.21 |  | 0.39 (0.29-0.52) | <0.001* |  | 0.64 (0.49-0.85) | 0.002* |  | 0.61 (0.47-0.79) | <0.001* |
|  | Linear trend |  |  |  |  |  | <0.001* |  |  | 0.003* |  |  | <0.001* |
| **Men** ^a^ | Low at BL & FU | 2860 | 100 | 3.5 |  | 1 (Ref) | NA |  | 1 (Ref) | NA |  | 1 (Ref) | NA |
|  | High at BL or FU only | 4297 | 115 | 2.68 |  | 0.70 (0.49-1.01) | 0.059 |  | 0.77 (0.53-1.09) | 0.137 |  | 0.71 (0.46-1.09) | 0.108 |
|  | High at BL & FU | 2802 | 62 | 2.21 |  | 0.65 (0.43-0.99) | 0.047* |  | 0.70 (0.48-1.02) | 0.064 |  | 0.63 (0.44-0.90) | 0.011* |
|  | Linear trend |  |  |  |  |  | 0.032* |  |  | 0.041* |  |  | 0.008* |
| **Women** ^a^ | Low at BL & FU | 1626 | 35 | 2.15 |  | 1 (Ref) | NA |  | 1 (Ref) | NA |  | 1 (Ref) | NA |
|  | High at BL/FU only | 7139 | 85 | 1.19 |  | 0.51 (0.34-0.77) | 0.001* |  | 0.76 (0.50-1.16) | 0.205 |  | 0.82 (0.54-1.26) | 0.366 |
|  | High at BL & FU | 9333 | 85 | 0.91 |  | 0.40 (0.26-0.61) | <0.001* |  | 0.60 (0.38-0.94) | 0.025* |  | 0.63 (0.41-0.98) | 0.041* |
|  | Linear trend |  |  |  |  |  | <0.001* |  |  | 0.026* |  |  | 0.030* |

Model 1: Unadjusted

Model 2: Adjusted for age, sex, ethnicity

Model 3: Adjusted for age, sex, ethnicity, socioeconomic status (housing type), education, brisk walking and sports activities at baseline, nutritional risk, smoking, alcohol, marital status

^a^ Sex was excluded from model adjustments

BL=Baseline, FU=Follow-up, MET=Metabolic equivalent of task, Ref=Reference hazard ratio of 1.00, HR=Hazard Ratio, CI=Confidence Interval; *Statistically significant at p<0.05

| **eTable 3.** Associations of participation in housework dichotomised by top quartile duration (min/week) with all-cause mortality | | | | | | | | | | | | | |
| --- | --- | --- | --- | --- | --- | --- | --- | --- | --- | --- | --- | --- | --- |
|  |  | Exposed | Deaths | *Per 100 person-years* |  | Model 1 |  |  | Model 2 |  |  | Model 3 |  |
|  |  | *person-years* | *N* |  |  | HR (95%CI) | p value |  | HR (95%CI) | p value |  | HR (95%CI) | p value |
| ***Light housework (cut-off ≥840 min/week)*** | | | |  |  |  |  |  |  |  |  |  |  |
| **Total** | Low at BL & FU | 12322 | 325 | 2.64 |  | 1 (Ref) | NA |  | 1 (Ref) | NA |  | 1 (Ref) | NA |
|  | High at BL/FU only | 11374 | 127 | 1.12 |  | 0.44 (0.29-0.67) | 0.002* |  | 0.61 (0.43-0.87) | 0.011* |  | 0.64 (0.42-0.95) | 0.032* |
|  | High at BL & FU | 4645 | 38 | 0.82 |  | 0.28 (0.14-0.59) | 0.004* |  | 0.46 (0.21-1.01) | 0.053 |  | 0.46 (0.21-0.98) | 0.046* |
|  | Linear trend |  |  |  |  |  | 0.003* |  |  | 0.023* |  |  | 0.028* |
| **Men** ^a^ | Low at BL & FU | 6418 | 221 | 3.44 |  | 1 (Ref) | NA |  | 1 (Ref) | NA |  | 1 (Ref) | NA |
|  | High at BL or FU only | 3131 | 49 | 1.57 |  | 0.48 (0.28-0.82) | 0.012* |  | 0.55 (0.35-0.86) | 0.012* |  | 0.55 (0.33-0.94) | 0.031* |
|  | High at BL & FU | 561 | 14 | 2.5 |  | 0.54 (0.21-1.39) | 0.183 |  | 0.61 (0.23-1.59) | 0.284 |  | 0.57 (0.23-1.45) | 0.220 |
|  | Linear trend |  |  |  |  |  | 0.029* |  |  | 0.041* |  |  | 0.057 |
| **Women** ^a^ | Low at BL & FU | 5904 | 104 | 1.76 |  | 1 (Ref) | NA |  | 1 (Ref) | NA |  | 1 (Ref) | NA |
|  | High at BL/FU only | 8243 | 78 | 0.95 |  | 0.56 (0.36-0.89) | 0.017* |  | 0.69 (0.46-1.04) | 0.071 |  | 0.72 (0.44-1.18) | 0.171 |
|  | High at BL & FU | 4084 | 24 | 0.59 |  | 0.34 (0.14-0.81) | 0.021* |  | 0.42 (0.17-1.01) | 0.053 |  | 0.43 (0.18-1.02) | 0.055 |
|  | Linear trend |  |  |  |  |  | 0.018* |  |  | 0.041* |  |  | 0.048* |
| ***Heavy housework (cut-off ≥60 min/week)*** | | | | |  |  |  |  |  |  |  |  |  |
| **Total** | Low at BL & FU | 17033 | 355 | 2.08 |  | 1 (Ref) | NA |  | 1 (Ref) | NA |  | 1 (Ref) | NA |
|  | High at BL or FU only | 10045 | 122 | 1.21 |  | 0.56 (0.44-0.71) | <0.001* |  | 0.75 (0.58-0.95) | 0.021* |  | 0.71 (0.56-0.91) | 0.007* |
|  | High at BL & FU | 1504 | 20 | 1.33 |  | 0.65 (0.23-1.87) | 0.362 |  | 0.91 (0.31-2.68) | 0.844 |  | 0.86 (0.23-3.20) | 0.776 |
|  | Linear trend |  |  |  |  |  | 0.040* |  |  | 0.333 |  |  | 0.287 |
| **Men** ^a^ | Low at BL & FU | 6682 | 215 | 3.22 |  | 1 (Ref) | NA |  | 1 (Ref) | NA |  | 1 (Ref) | NA |
|  | High at BL/FU only | 3058 | 62 | 2.03 |  | 0.62 (0.45-0.84) | 0.002* |  | 0.74 (0.52-1.04) | 0.081 |  | 0.71 (0.50-0.99) | 0.044* |
|  | High at BL & FU | 363 | 6 | 1.65 |  | 0.71 (0.14-3.58) | 0.628 |  | 0.81 (0.18-3.65) | 0.754 |  | 0.80 (0.14-4.65) | 0.771 |
|  | Linear trend |  |  |  |  |  | 0.114 |  |  | 0.297 |  |  | 0.277 |
| **Women** ^a^ | Low at BL & FU | 10351 | 140 | 1.35 |  | 1 (Ref) | NA |  | 1 (Ref) | NA |  | 1 (Ref) | NA |
|  | High at BL or FU only | 6987 | 60 | 0.86 |  | 0.59 (0.42-0.84) | 0.004* |  | 0.75 (0.53-1.05) | 0.096 |  | 0.72 (0.51-1.00) | 0.051 |
|  | High at BL & FU | 1140 | 14 | 1.23 |  | 0.78 (0.29-2.08) | 0.581 |  | 0.99 (0.33-2.96) | 0.992 |  | 0.91 (0.29-2.81) | 0.85 |
|  | Linear trend |  |  |  |  |  | 0.121 |  |  | 0.503 |  |  | 0.373 |

Model 1: Unadjusted

Model 2: Adjusted for age, sex, ethnicity

Model 3: Adjusted for age, sex, ethnicity, socioeconomic status (housing type), education, brisk walking and sports activities at baseline, nutritional risk, smoking, alcohol, marital status

^a^ Sex was excluded from model adjustments

BL=Baseline, FU=Follow-up, Ref=Reference hazard ratio of 1.00, HR=Hazard Ratio, CI=Confidence Interval; *Statistically significant at p<0.05
